# Retraction: Indole-3-Carbinol Inhibits Nasopharyngeal Carcinoma Growth through Cell Cycle Arrest *In Vivo* and *In Vitro*

**DOI:** 10.1371/journal.pone.0275881

**Published:** 2022-10-04

**Authors:** The PLOS ONE Editors

After this article [1] was published, concerns were raised about the mouse tumor sizes reported in Fig. 7. Specifically:

- The charts in Fig. 7A and 7B of the article appear to report tumor sizes of up to 3000 mm^3^.
- Based on the mouse images in Fig. 7C, particularly the third, fifth and sixth mice, it appears as though the tumor sizes could impede mobility and ulceration and necrosis are visible.

In response to queries about these experiments, the corresponding author stated that when this study was conducted China did not strictly regulate animal research ethics, the author’s institution did not seem to have clear regulations pertaining to animal tumor sizes, and the study underwent only a ‘simple’ ethical review. They selected eight weeks as the end point of the experiment, at which time the experimental animals were anesthetized with ether and then killed. They also stated that during the experimental period, they measured the body weight and food intake of the animals on a daily basis and made efforts to provide adequate nutrition. A copy of the ethics approval letter for the study was provided. The corresponding author stated that the underlying individual-level tumor volume and mouse weight data are not available.

The *PLOS ONE* Editors consulted with an expert in laboratory animal welfare who assessed the article and the authors’ comments and confirmed that the tumor appearances and sizes reported in this article would likely have impeded mobility and caused significant pain and discomfort. Based on the results shown in Fig. 7 the expert advised that this study would not have met U.S. laboratory research standards for humane endpoints in 2013.

Adding to the animal ethics and welfare concerns, quantitative data reported in Fig. 7A, 7B indicate that the 8-week endpoint was likely not scientifically justified: it appears there were likely significant differences between groups by 4–6 weeks, when tumors were <2000 mm^3^ in volume.

*PLOS ONE* is also concerned about the use of ether as an anesthetic agent, since this agent can cause irritation and distress for laboratory animals and also presents risks to laboratory personnel.

In light of the above concerns, *PLOS ONE* concluded that the study did not comply with the journal’s Animal Research Policy which requires that studies involving animals must have been conducted according to internationally-accepted standards. Therefore, *PLOS ONE* retracts this article. The editors regret that these concerns were not addressed prior to the article’s publication.

ZZT responded but expressed neither agreement nor disagreement with the editorial decision. ZC, SMC, CC, FL and BKX either did not respond directly or could not be reached.
